# Longitudinal plasma protein profiling of newly diagnosed type 2 diabetes

**DOI:** 10.1016/j.ebiom.2020.103147

**Published:** 2020-12-03

**Authors:** Anders Gummesson, Elias Björnson, Linn Fagerberg, Wen Zhong, Abdellah Tebani, Fredrik Edfors, Caroline Schmidt, Annika Lundqvist, Martin Adiels, Fredrik Bäckhed, Jochen M Schwenk, Per-Anders Jansson, Mathias Uhlén, Göran Bergström

**Affiliations:** a Department of Molecular and Clinical Medicine, Institute of Medicine, Sahlgrenska Academy, Gothenburg University, Gothenburg, Sweden; b Department of Clinical Genetics and Genomics, Region Västra Götaland, Sahlgrenska University Hospital, Gothenburg, Sweden; c Science for Life Laboratory, Department of Protein Science, KTH-Royal Institute of Technology, Stockholm, Sweden; d Novo Nordisk Foundation Center for Basic Metabolic Research, Section for Metabolic Receptology and Enteroendocrinology, Faculty of Health Sciences, University of Copenhagen, Copenhagen, Denmark; e Department of Clinical Physiology, Region Västra Götaland, Sahlgrenska University Hospital, Gothenburg, Sweden; f Department of Neuroscience, Karolinska Institutet, Stockholm, Sweden

**Keywords:** Type 2 diabetes, Plasma proteomics, Longitudinal profiling, Precision medicine

## Abstract

**Background:**

Comprehensive proteomics profiling may offer new insights into the dysregulated metabolic milieu of type 2 diabetes, and in the future, serve as a useful tool for personalized medicine. This calls for a better understanding of circulating protein patterns at the early stage of type 2 diabetes as well as the dynamics of protein patterns during changes in metabolic status.

**Methods:**

To elucidate the systemic alterations in early-stage diabetes and to investigate the effects on the proteome during metabolic improvement, we measured 974 circulating proteins in 52 newly diagnosed, treatment-naïve type 2 diabetes subjects at baseline and after 1 and 3 months of guideline-based diabetes treatment, while comparing their protein profiles to that of 94 subjects without diabetes.

**Findings:**

Early stage type 2 diabetes was associated with distinct protein patterns, reflecting key metabolic syndrome features including insulin resistance, adiposity, hyperglycemia and liver steatosis. The protein profiles at baseline were attenuated during guideline-based diabetes treatment and several plasma proteins associated with metformin medication independently of metabolic variables, such as circulating EPCAM.

**Interpretation:**

The results advance our knowledge about the biochemical manifestations of type 2 diabetes and suggest that comprehensive protein profiling may serve as a useful tool for metabolic phenotyping and for elucidating the biological effects of diabetes treatments.

**Funding:**

This work was supported by the Swedish Heart and Lung Foundation, the Swedish Research Council, the Erling Persson Foundation, the Knut and Alice Wallenberg Foundation, and the Swedish state under the agreement between the Swedish government and the county councils (ALF-agreement).

Research in Context

**Evidence before this study**

Circulating protein signatures may provide important information about the molecular phenotype of diabetes and the cardiometabolic health state of individuals. Our knowledge about the protein alterations of diabetes have grown considerably over the recent years, however this knowledge is mainly based on cross-sectional data from patients with different durations of the disease and with ongoing diabetes treatment.

**Added value of this study**

This study adds to previous proteomic studies since it describes the protein signatures of newly diagnosed, treatment naive type 2 diabetes and investigates the relative importance of diabetes-related metabolic features for these signatures. Most importantly, the study adds the longitudinal aspect by performing repeated plasma profiling during standard diabetes treatment so that the dynamics during metabolic improvement can be elucidated. The comprehensive protein analyses revealed previously unknown associations with diabetes, as well as confirming previously published associations, thus contributing to our knowledge about the biochemical manifestations of diabetes.

**Implications of all the available evidence**

A broad range of blood-borne proteins are altered in newly diagnosed type 2 diabetes and protein profiling show promising potential as a cardiometabolic health indicator. In addition, protein patterns are sensitive to changes in metabolic status as well as to metformin medication, indicating that protein profiling can help to elucidate the molecular effects of diabetes treatments.

Alt-text: Unlabelled box

## 1 Introduction

Type 2 diabetes, characterized by hyperglycemia on account of chronic insulin resistance and impaired pancreatic β-cell function, is a complex systemic disease with dysregulated metabolic pathways and complications in several organ systems. The early stage of the disease frequently goes undiagnosed for many years because hyperglycemia develops gradually and is often not severe enough for the patient to notice the classic symptoms of diabetes [1]. Despite this seemingly mild disease status, many of the pathophysiological processes of diabetes-related complications are already present, partly due to hyperglycemia, but also due to the other cardiometabolic risk factors that commonly accompany type 2 diabetes such as obesity, hypertension, dyslipidemia and non-alcoholic fatty liver disease (NAFLD) [2].

The onset of type 2 diabetes involves numerous pathways and interactions between metabolically active tissues such as pancreas, liver, gut, adipose tissue and skeletal muscle [3]. Many of these interactions are mediated through various circulating proteins, including hormones, growth factors, adipokines, cytokines and enzymes [4]. The recent advancements in high throughput technologies for measuring a large number of proteins in a single assay have enabled data-driven discoveries that may offer new insights into the dysregulated metabolic milieu of diabetes [5,6]. There is also a potential for comprehensive protein profiling in personalized medicine, by detecting early signs of disease development and providing simultaneous information on multiple cardiometabolic health indicators in individual patients [7]. In addition, protein profiling during diabetes treatments such as diet, physical activity and pharmacotherapy could potentially help to broaden our understanding of the therapeutic mechanisms [8].

While efforts have been made to study protein alterations in diabetes [5,6], little is known about proteomic alterations at the very onset of the disease and before any diabetes treatment has been initiated. Patients at this stage of the disease can only be reached using screening programs since they lack classic symptoms of diabetes. Furthermore, there is limited information about the relative importance of hyperglycemia versus other metabolic aberrations for the protein signatures in blood. Identifying the main cardiometabolic drivers of protein patterns in blood has implications not only for the basic understanding of diabetes, but also for the potential of protein profiling as a cardiometabolic health indicator in these patients. Therefore, proteomic profiling could be a future approach to monitor diabetes interventions and it is therefore of major interest to understand to what extent the protein signatures of diabetes subjects are sensitive to the clinical improvement that occur during diabetes treatment.

Recently, we have conducted a large research program to analyze “wellness” in the general population involving the molecular phenotypes of a longitudinal cohort, the Swedish SciLifeLab SCAPIS Wellness Profiling (S3WP) program. This has led to several articles regarding “wellness”, including Zhong et al., [9], Dodig-Crnkovic et al. [10] and Tebani et al. [11]. Here, we have used the same approach to target type 2 diabetes and describe for the first time a comprehensive analysis of plasma protein profiles in newly diagnosed type 2 diabetes patients before and after diabetes treatment, while comparing their protein profiles to that on non-diabetes controls.

Fifty-two individuals with previously undiagnosed and treatment-naïve type 2 diabetes were identified from large population-based screening programs and selected for a longitudinal study. Plasma protein profiles, based on 974 unique proteins, were analyzed using targeted affinity proteomics. The protein profiles were analyzed at baseline and after one and three months of guideline-based diabetes treatment, and the protein profiles of the diabetes group were compared to that of 94 subjects without diabetes in the S3WP program. In this way, we were able to conduct a comprehensive protein profiling to unveil systemic alterations of early-stage of diabetes and to investigate effects on the proteome during glucose lowering treatment.

## 2 Methods

### 2.1 Study design and subjects

The diabetes group consisted of 52 subjects, age 50–65 years, with no history of diabetes who were diagnosed during population-based screening examinations at the Sahlgrenska University Hospital, Gothenburg, and consecutively invited to the current study. The diagnosis was based on fasting p-glucose and oral glucose tolerance tests (OGTT). Presence of diabetes was defined according the Swedish standard, corresponding to the American Diabetes Association standards [1]: A fasting p-glucose ≥7.0 mmol/L or an 2-hour OGTT p-glucose ≥11.1 mmol/L (≥12.2 mmol/L when measured capillary). Subjects who met diabetes criteria were scheduled for a second glucose measurement on a separate occasion and enrolled if diabetes diagnosis was confirmed. To identify latent autoimmune diabetes in adults (LADA), glutamic acid decarboxylase (GAD), tyrosine phosphatase IA-2 (IA-2) and zinc transporter 8 (ZnT8) antibodies were measured. Exclusion criteria were severe hyperglycemia requiring hospitalization or immediate insulin treatment, presence of any clinically significant disease which, in the opinion of the investigator, may interfere with the subject´s ability to participate in the study, or any major surgical procedure or trauma within four weeks of the first study visit. The diabetes group was examined at baseline and after one and three months of guideline-based diabetes treatment according to first-line therapy with lifestyle change including weight management and physical activity, with or without metformin as judged by the treating physician. Of the 52 subjects, 51 (98%) completed the 3-month follow-up visit. The non-diabetes control group consisted of 94 participants that completed the second year in the longitudinal Swedish SciLifeLab SCAPIS Wellness Profiling (S3WP) program [[9], [10], [11]] and did not have diabetes as judged from repeated fasting glucose and HbA1c measurements as well as baseline OGTT. The control group were examined twice during the same time-period and at the same site as the diabetes subjects (2016–2018, Wallenberg Laboratory, Gothenburg), and the mean values from these examinations were used in the data analysis.

### 2.2 Ethics

The study conforms to the ethical guidelines of the 1975 Declaration of Helsinki and was approved by the Ethical Review Board of Gothenburg, Sweden (DNR 448-16, 407-15). All participants provided written informed consent.

### 2.3 Clinical data

All study visits were performed after an overnight fast of at least 8 h. Study assessments in both groups included anthropometry, blood pressure, clinical chemistry and life-style questionnaires. Weight was measured with participants in light clothing using calibrated scales, and the body mass index (BMI) was calculated by dividing the weight (kg) by the square of the height (m). Waist circumference was measured midway between the palpated iliac crest and the palpated lowest rib margin in the left and right mid-axillary lines. Total body fat was measured using a bioelectrical impedance scale (Tanita MC780MA, Tanita Corporation, Tokyo, Japan) according to manufacturer´s instructions. Systolic and diastolic blood pressure (SBP, DBP) was registered in supine position and after 5 min of rest, using the automatic Omron P10. Clinical chemistry and hematology measurements included fasting glucose, hemoglobin A1c (HbA1c), low-density lipoprotein cholesterol (LDL-C), high-density lipoprotein cholesterol (HDL-C), triglycerides (TG), apolipoprotein A1 (ApoA1), apolipoprotein B (ApoB), creatinine, high sensitive C-reactive protein (CRP), alanine aminotransferase (ALAT), gamma glutamyl transferase (GGT), urate, cystatin C, N-terminal pro-brain natriuretic peptide (NT-proBNP), hemoglobin (Hb), white blood cell count (WBC), red blood cell count (RBC) and platelet count. Estimated glomerular filtration rate (eGFR) was calculated from age, gender, creatinine and cystatin C according to the Chronic Kidney Disease Epidemiology Collaboration (CKD-EPI) 2012 formula [12]. Insulin and C-peptide was measured in the diabetes group and the homeostatic model assessment of insulin resistance (HOMA-IR) was calculated according to the formula: fasting insulin (mU/L) x fasting glucose (mmol/L) / 22.5 [13]. Baseline measurements of liver fat content and visceral adipose tissue area (VAT) were performed in the diabetes group using a dedicated dual-source CT scanner equipped with a Stellar Detector (Siemens, Somatom Definition Flash, Siemens Medical Solution, Forchheim, Germany) as previously described [14].

### 2.4 Plasma protein measurements

All plasma samples were collected after an overnight fast and at the same visit as the clinical examinations. For three subjects, plasma samples for protein measurements were not available from the 1-month visit. Multiplex proximity extension assays (PEA, Olink Bioscience, Uppsala, Sweden) were used to measure the relative concentrations of plasma proteins. Each kit provides a microtiter plate for measuring 92 protein biomarkers in all prepared samples. Each well contains 96 pairs of DNA-labeled antibody probes. Samples were incubated in the presence of proximity antibody pairs tagged as previously described [15]. To minimize inter- and intra-run variation, samples from the diabetes and control group were mixed and randomized across plates. Both internal control (extension control) and inter-plate control were used for normalization and then transformed using a pre-determined correction factor. The pre-processed data were provided in the arbitrary unit Normalized Protein eXpression (NPX) on a log2 scale, where a high NPX value represents high protein concentration. The analyses were performed at SciLifeLab's Plasma Profiling facility on eleven Olink panels including Cardiometabolic, Cell Regulation, Cardiovascular II, Cardiovascular III, Development, Immune Response, Oncology II, Inflammation, Metabolism, Neurology, and Organ Damage. Quality control was performed at both sample and protein levels and resulted in using a total of 974 unique proteins in 340 samples.

The validation of epithelial cell adhesion molecule (EPCAM) was analysed in EDTA plasma diluted 1:3 using human EPCAM ELISA Kit (ab155442, Abcam, Cambridge, GB). Samples below detection limit were considered as 50% of the sensitivity of the ELISA (22,5 pg/ml) for statistical analysis.

### 2.5 Statistics

R version 3.6.1 was used for all statistical analyses. Imputation of protein abundances were performed using the function rfImpute in the package randomForest [16]. A protein was not imputed but instead excluded from the analysis if more than 20 percent of the values were missing (which was the case for *n* = 1 protein). In total, less than 0.2% of the data was imputed. To study the overall protein profile of newly diagnosed type 2 diabetes, we applied linear discriminant analysis (LDA) on the proteomic dataset from the diabetes group´s baseline visit and the control group to maximize the component axes for group separation. We subsequently applied the LDA to identify diabetes status from the proteomic data, and to test robustness of the LDA model we also used prediction models based on both random forest and support vector machine learning since these methods are well established and represent different approaches to prediction. Linear discriminant analysis (LDA) was performed using the package “MASS” in R [17], random forest prediction modeling using “randomForest” [16], and support vector machines using the “e1071” [18] with the default radial kernel and the default parameter settings. Training set (50%) and test set (50%) were utilized to evaluate the performance based on the area under the receiver operating characteristic curve (AUROC) of the machine learning algorithms. Relative importance analysis was performed using the package “relaimpo” with the method “lmg” [19], and variable importance ranks (Gini coefficients and accuracy decreases) using “randomForestExplainer” [20]. Correlation coefficients refer to Spearman's rank correlation coefficients, and p-values were calculated using the Mann-Whitney U test for non-paired samples or the Wilcoxon signed-rank test for paired samples. Effect sizes were calculated from the Z-scores and the sample sizes of the respective significance tests. Mixed-modeling was performed using the package “lme4” [21] with metformin dose and visit (which adjusts for other effects related to the intervention) as random effects and subject as fixed effect. The control group was only used to determine the diabetes-associated protein profile and all other analyses were performed within the diabetes group to minimize the risk of bias being carried over from the group-wise comparisons. To correct for multiple comparisons, p-values were adjusted to a false discovery rate (FDR) of 0.05, based on the total number of proteins studied (i.e. 974).

### 2.6 Role of funding source

The funders did not have any role in study design, data collection, data analyses, interpretation, or writing of report.

## 3 Results

### 3.1 Clinical characteristics at baseline

Screening for diabetes was done in ongoing population studies at the Sahlgrenska University Hospital. The frequency of newly diagnosed diabetes based on repeated fasting capillary plasma glucose measurements and 2-hour OGTT was 1.7% and 0.5%, respectively. Of the 52 subjects that were included in the diabetes group, 35 (67%) had fasting glucose ≥7.0 at two separate occasions before inclusion, and for the remaining 17 subjects the 2-hour OGTT was required for diagnosis on at least one occasion. In addition to having higher fasting glucose and HbA1c, the diabetes group differed from the control group regarding the classical features of the metabolic syndrome, i.e. the diabetes group was more obese, had higher blood pressure, higher serum triglycerides and lower serum HDL levels. Liver function tests and inflammatory markers were also increased (Table 1).

**Table 1:** Comparison of clinical characteristics of diabetes group and control group.

Abbreviation	Clinical variable	Diabetes (*n* = 52)	Control (*n* = 94)
Gender	Males, n (%)	21 (40)	47 (50)
Age	Age, years	59.9 (8.4)	58.2 (7.0)
Smoking	Current smoker, n (%)	9 (17)	1 (1)
SedentaryTime	Sedentary time, hours	8.0 (5.0)	6.5 (4.0)
BMI	Body mass index, kg/m^2^	31.9 (9.9)*	25.1 (5.1)
Waist	Waist circumference, cm	108.5 (25.3)*	93.8 (14.9)
Bodyfat	Body fat content,%	32.4 (13.2)*	25.0 (12.9)
SBP	Systolic blood pressure, mmHg	131.5 (23.0)*	119.0 (20.1)
DBP	Diastolic blood pressure, mmHg	86.0 (13.5)*	78.0 (12.4)
Gluc	Glucose, mmol/L	7.5 (1.6)*	5.7 (0.7)
HbA1c	Hemoglobin A1c, mmol/mol	43.0 (7.5)*	34.3 (3.9)
LDL-C	Low density lipoprotein cholesterol, mmol/L	3.3 (1.0)†	3.6 (0.9)
HDL-C	High density lipoprotein cholesterol, mmol/L	1.4 (0.5)*	1.8 (0.7)
TG	Triglycerides, mmol/L	1.5 (0.6)*	0.9 (0.5)
ApoA1	Apolipoprotein A1, g/L	1.5 (0.3)*	1.7 (0.4)
ApoB	Apolipoprotein B, g/L	1.0 (0.3)	1.1 (0.2)
ALAT	Alanine aminotransferase, µkat/L	0.55 (0.36)*	0.39 (0.17)
GGT	Gamma glutamyltransferase, µkat/L	0.62 (0.55)*	0.32 (0.21)
NT-proBNP	N-terminal pro b-type natriuretic peptide, ng/L	43.5 (59.8)	46.3 (58.4)
eGFR	Estimated glomerular filtration rate, mL/min/1.73 m^2^	77.4 (13.0)†	86.0 (15.3)
Urate	Urate, µmol/L	363.5 (114.3)*	291.0 (83.4)
CRP	C-reactive protein, high sensitivity, mg/L	2.6 (3.6)*	0.9 (1.4)
WBC	White blood cell count, x10^9^/L	5.8 (2.3)†	5.0 (1.4)
Hb	Hemoglobin, g/L	147.0 (14.3)†	143.0 (14.4)
RBC	Red blood cell count, x10^12^/L	4.8 (0.4)†	4.6 (0.5)
Platelets	Platelet count, x10^9^/L	210.5 (68.8)*	238.0 (76.6)

Values are median (interquartile range) unless otherwise indicated. The symbol * denotes *p*<0.001 and † denotes *p*<0.01 [Mann-Whitney U test].

### 3.2 Protein profiles at baseline

**Fig. 1:**
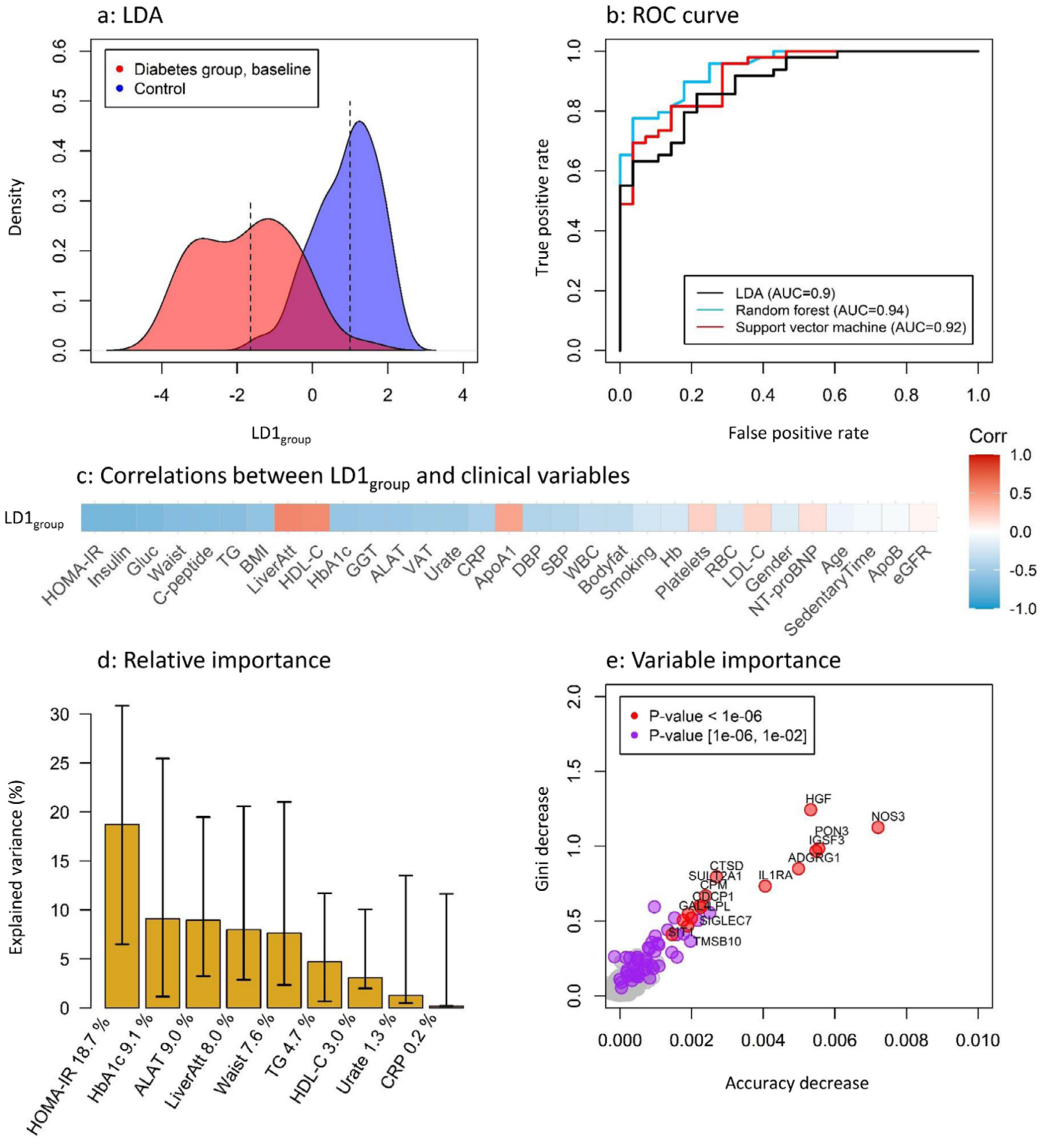
(a) Density plot of the first latent dimension from the Linear Discriminant Analysis (LDA) based on the proteomic data. This plot shows the separation trend between the diabetes and control groups along the linear discriminant dimension 1 (LD1_group_) which is a one-dimensional representation of the proteomic group differences. (b) Three machine learning algorithms (LDA, Random forest and support vector machine) were benchmarked to evaluate the classification performance of the protein signature to discriminate diabetes subjects from controls. The algorithms were trained on half of the cohort and internally validated on half of the cohort. Classification accuracy is presented as area under the curve (AUC) of the receiver operating characteristics (ROC) from the validation sample. (c) LD1_group_ correlated against clinical variables within the diabetes group. The order of clinical variables is based on the strength of correlation, the highest correlation shown to the left. Note that a low liver attenuation value corresponds to high liver fat content. (d) Relative importance analysis of clinical variables reveal that HOMA-IR explains most of the independent variability in LD1_group_, followed by HbA1c, ALAT, liver fat and waist circumference. Insulin, glucose, BMI/VAT and GGT were excluded from the analysis due to high covariance with HOMA-IR, HbA1c, waist and ALAT, respectively. (e) Variable importance for the random forest diabetes classification model reveals NOS3, HGF, PON3, IGSF3 and ADGRG1 as the strongest predictors of diabetes status.

To investigate the protein signature of the diabetes group, LDA was applied to determine the first linear discriminant for group separation (LD1_group_). The LDA showed that the overall protein profile clearly differed between the diabetes group´s baseline visit and the control group (Fig 1a). When LDA, random forest and support vector machine were used to build prediction models based on the overall proteome, all three methods were able to identify diabetes status from the protein signature. The AUROCs were 0.90 (CI: 0.83–0.97), 0.94 (CI: 0.90–0.99) and 0.92 (CI: 0.86–0.98) for LDA, random forest and support vector machine learning, respectively (Fig. 1b). Variability in LD1_group_ correlated with several features of the metabolic syndrome including insulin resistance (HOMA-IR), insulin homeostasis (insulin, C-peptide), NAFLD (Liver fat, GGT, ALAT), glucose control (fasting glucose, HbA1c) and obesity (waist circumference, BMI), high triglycerides, and low HDL-C (Fig 1c). In a relative importance analysis that included variables with the highest correlations with LD1_group_, HOMA-IR remained most important for variance in LD1_group_ (Fig 1d). In total there were 44 out of 973 (4.5%) proteins that contributed significantly to the prediction of diabetes (FDR-corrected *p*<0.05) in the random forest model, the most important being NOS3, HGF, PON3, IGSF3, and ADGRG1 (Fig 1e).

**Fig. 2:**
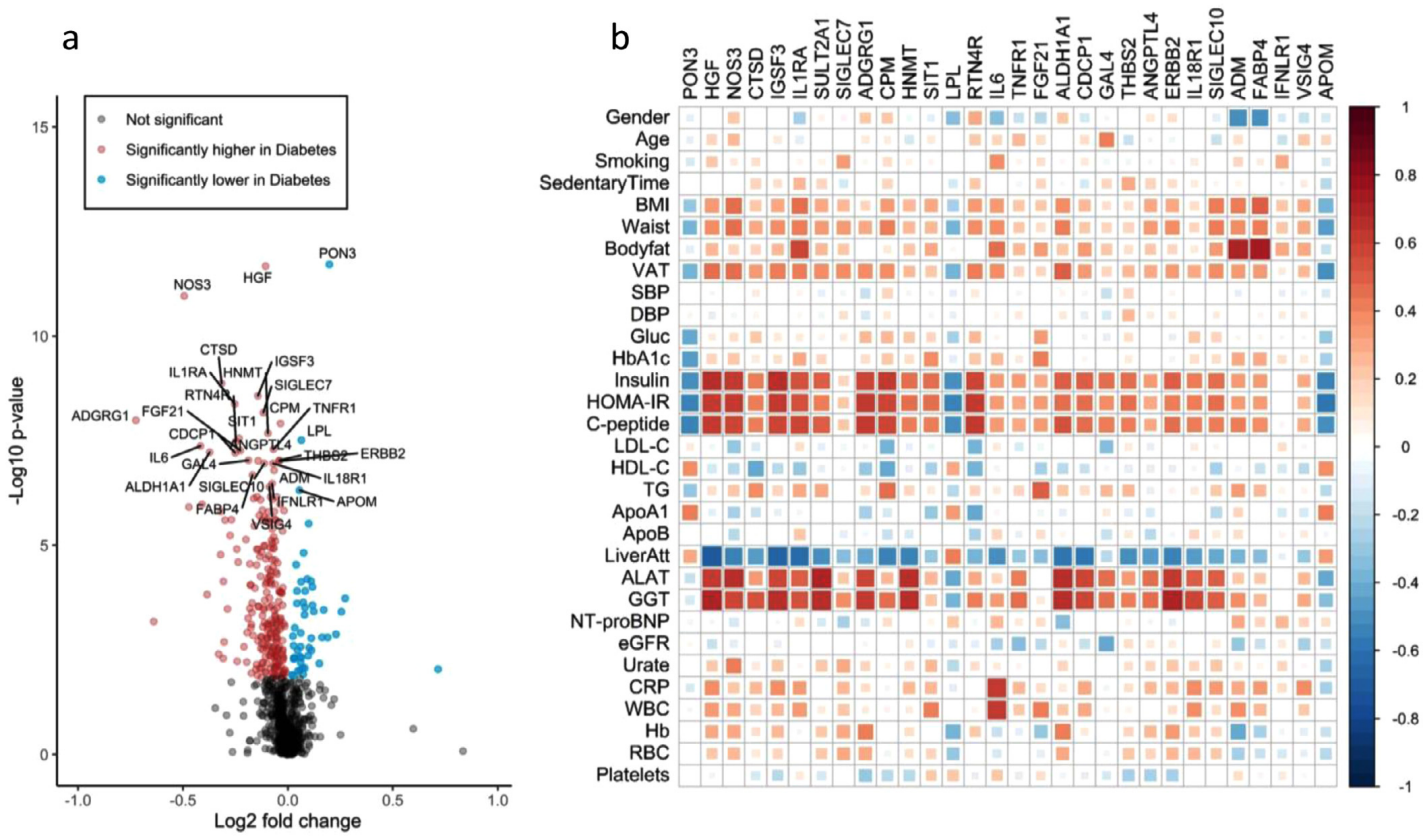
(a) Comparisons of plasma protein levels between the diabetes group and the control group (Mann-Whitney U test), visualized with a volcano plot with labels on the top 30 most significant proteins. (b) Correlation matrix of the top 30 diabetes-associated proteins and clinical variables in the diabetes group with red color representing positive and blue representing negative r-values. (For interpretation of the references to color in this figure legend, the reader is referred to the web version of this article.)

To identify plasma proteins that are altered in early-stage diabetes, we compared the plasma levels of each protein in the diabetes and control group using Mann-Whitney U test and found that 293 (30%) of the 974 proteins differed significantly between groups (Fig. 2a). The three proteins with the lowest p-value in the group comparison were PON3, HGF and NOS3 (FDR-corrected *p*<10^−8^). The top 30 most significant proteins from the group-wise comparison are listed in Supplemental Table S1 along with a brief summary of their implication in cardiometabolic disease, and all 293 significant proteins are listed in Supplemental Table S2. Correlations between the top 30 proteins and clinical variables are visualized in Fig. 2b. The highest correlations were found with measures related to NAFLD (e.g. *r* = 0.70 for HGF versus liver fat content and *r* = 0.71 for ERBB2 versus GGT) and there was also a pattern of several proteins correlating with measures of insulin homeostasis (e.g. *r* = 0.65 for IGSF3 versus insulin and *r* = 0.62 for ADGRG1 versus C-peptide). Correlations with measures of hyperglycemia, adiposity, lipids, blood pressure and inflammation were generally weaker, exceptions being FABP4 and ADM which correlated with body fat content (*r* = 0.75 and *r* = 0.69, respectively), and IL6 which correlated with CRP (*r* = 0.63) and WBC (*r* = 0.62). All top 30 proteins were associated with diabetes independently of age and sex when examined in linear regression models, and all but one (FABP4) of the associations were also independent of BMI (Supplemental Table S1).

### 3.3 Metabolic improvement during diabetes treatment

**Fig. 3:**
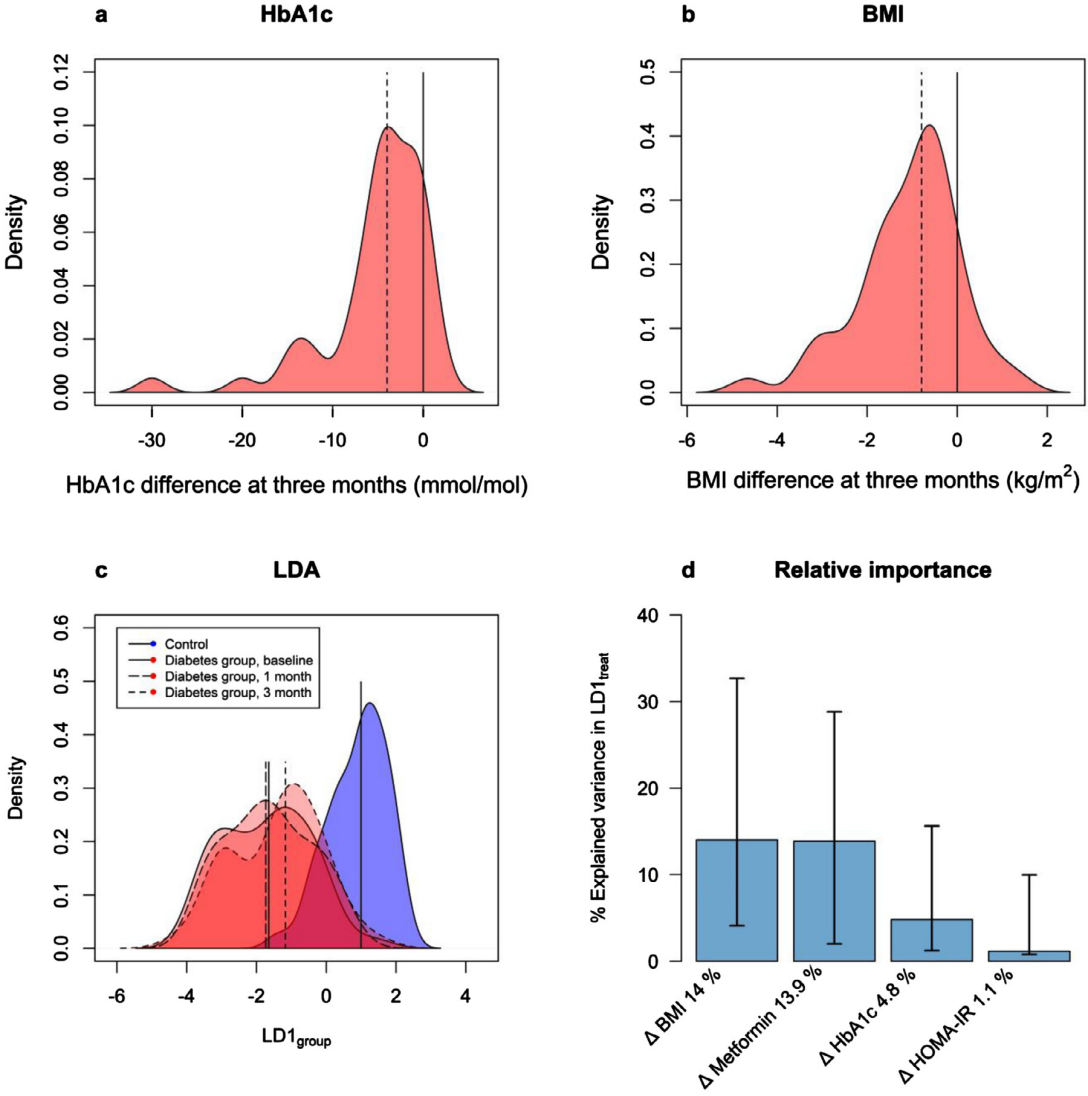
(a) Density plot of the change in HbA1c after 3 months in the diabetes group. The dashed line depicts the mean HbA1c reduction of 5.2 mmol/mol at visit 3. (b) Density plot of the change in BMI after 3 months in the diabetes group. The dashed line depicts the mean BMI reduction of 1.1 kg/m^2^. (c) Density plot of Linear Discriminant Analysis with the 1 and 3-month visit projected onto the LD1_group_-axis. The distributions between baseline and the 3-month visit are significantly different with the 3-month distribution being closer to the control group (*p*-value = 0.0092 [paired *t*-test], *n* = 51). (d) Relative importance analysis showing which changes in the clinical variables BMI, metformin treatment, HOMA-IR and HbA1c that explain the variation in the changing proteome during treatment (represented by change in LD1_treat_).

The diabetes subjects were followed over three months of guideline based diabetes treatment, and during this period the mean reduction in HbA1c was 5.2 mmol/mol and the mean weight loss 3.3 kg, corresponding to a mean BMI reduction of 1.1 kg/m^2^. Of the 51 subjects completing the study, 23 subjects had a HbA1c reduction of 5 mmol/mol or more and 22 subjects lost 3 kg or more in weight. The distributions of HbA1c and BMI-change are shown in Fig. 3a and 3b, respectively. There were also significant improvements in most of the other metabolic variables including blood pressure, serum lipids, liver function tests and CRP (Supplemental Table S3). At 3 months, 13 subjects had a low dose (0.5–1 g) of metformin and 29 had a high dose (1.5–2 g), whereas 9 subjects were not treated with metformin.

### 3.4 Changes in the plasma proteome during treatment

To test if the proteomic alterations of the diabetes group at baseline were attenuated during treatment, we used the previously determined first linear discriminant for group separation (LD1_group_) and analyzed how the diabetes subjects were distributed at the 3-month follow-up visit. The signatures of the diabetes group changed in the direction of the non-diabetic group during treatment and this shift in the distribution was significant at 3 months compared to baseline (paired *t*-test *p* = 0.0092) (Fig. 3c). To estimate the importance of improved glucose control, insulin sensitivity, weight loss and metformin medication for the proteomic variance during treatment, we determined the first linear discriminant of the comparison between baseline and 3 months of diabetes treatment (LD1_treat_) and performed a relative importance analysis (Fig. 3d). The results indicated that weight change and metformin medication had the largest importance, each explaining 14% of the proteome variance during treatment, whereas change in HbA1c and HOMA-IR only accounted for 4.8% and 1.1%, respectively.

**Fig. 4:**
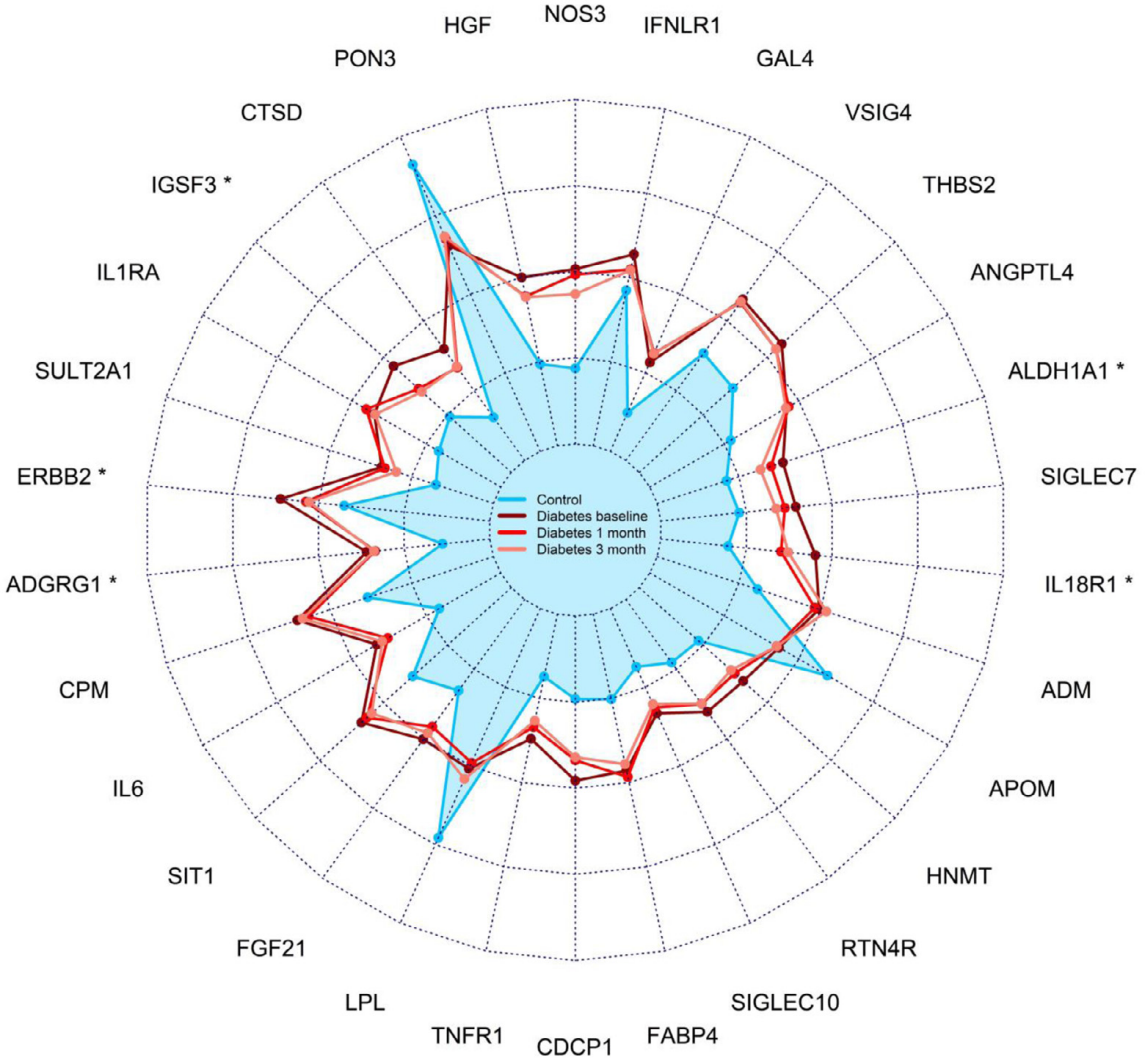
Radarplot showing levels of top 30 diabetes-associated proteins in the diabetes group at each timepoint and in the control group. The symbol * denotes FDR-corrected *p*<0.05 for change between baseline and 3 months in a paired Wilcoxon signed rank test.

**Fig. 5:**
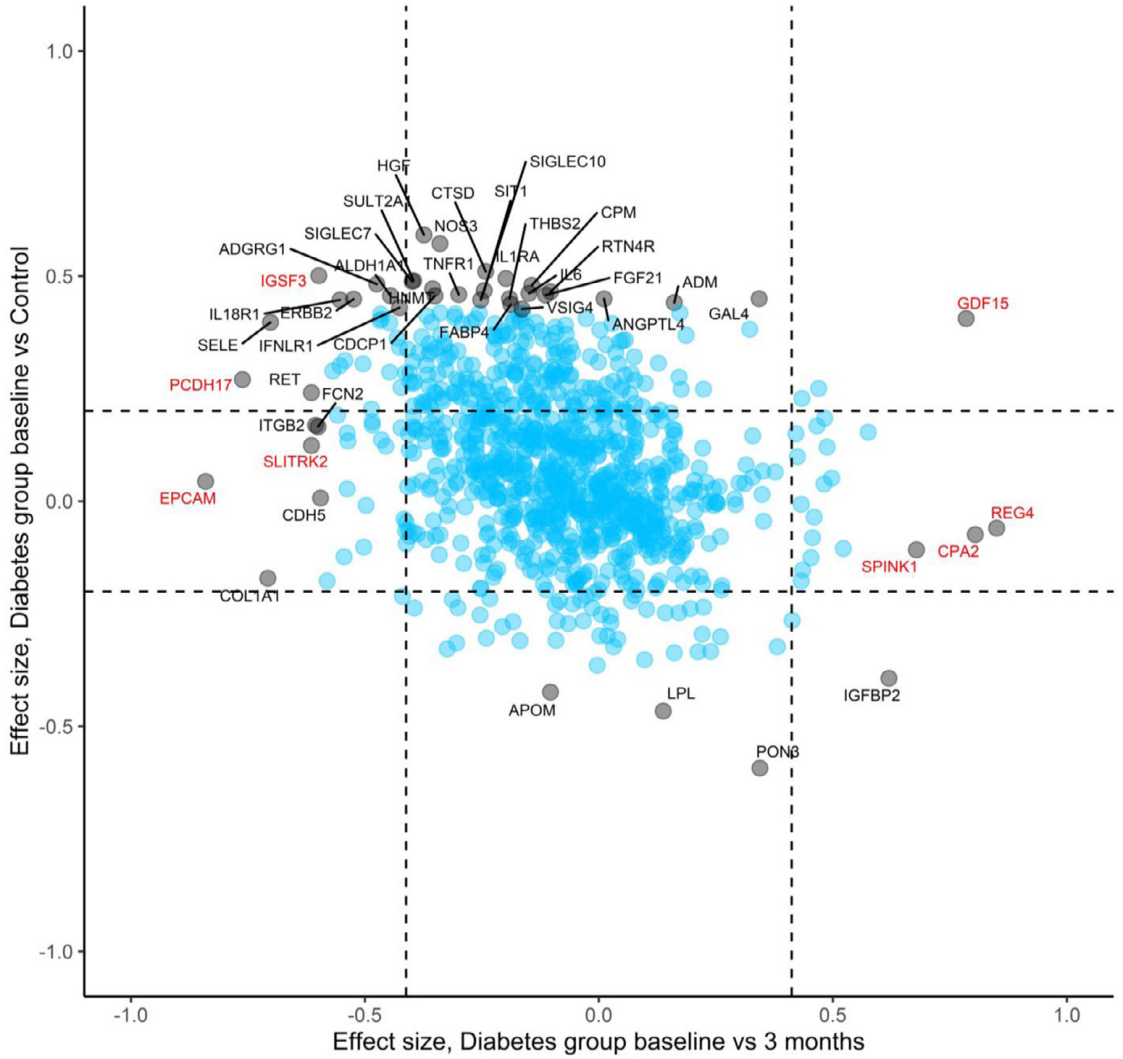
Plot showing effect sizes for all 974 proteins for the diabetes vs controls comparison (y-axis) and for changes during treatment in the diabetes group (x-axis). Dashed lines depict effect sizes corresponding to FDR-adjusted p-value of 0.05. The top 30 most significant proteins between groups at baseline are indicated with text, as is the top 15 most significantly changed proteins during treatment (some overlapping). Proteins also associated with metformin according to mixed model analysis are highlighted in red. (For interpretation of the references to color in this figure legend, the reader is referred to the web version of this article.)

Changes during treatment in the top 30 diabetes-associated proteins are shown in Fig. 4. Five (17%) of these proteins reached statistical significance for change from baseline in a FDR-adjusted paired Wilcoxon signed rank test, all changing in the direction of the control group. To visualize changes during treatment at a proteome level, we compared the 3-month visit with baseline for all proteins and plotted the effect-sizes in relation to the group-wise comparisons (Fig. 5). There was an overall trend towards normalization of the initial protein alterations during treatment, although only 22 (7.5%) of the 293 diabetes-associated proteins reached statistical significance for change from baseline. Notably, some of the most significant changes during treatment occurred in proteins that did not differ between groups at baseline. Furthermore, GDF15 showed a unique pattern with elevated levels in the diabetes group at baseline which were further increased during treatment. A mixed model analysis revealed that the five proteins presenting the most pronounced changes during treatment were all independently associated with metformin medication, including EPCAM (*p* = 2.6 × 10^−9^), GDF15 (*p* = 5.6 × 10^−8^), REG4 (*p* = 6.4 × 10^−5^), PCDH17 (*p* = 2.6 × 10^−4^) and CPA2 (*p* = 6.7 × 10^−4^) (Fig 5 and Supplemental Table S4).

### 3.5 Validation of the metformin-EPCAM association

**Fig. 6:**
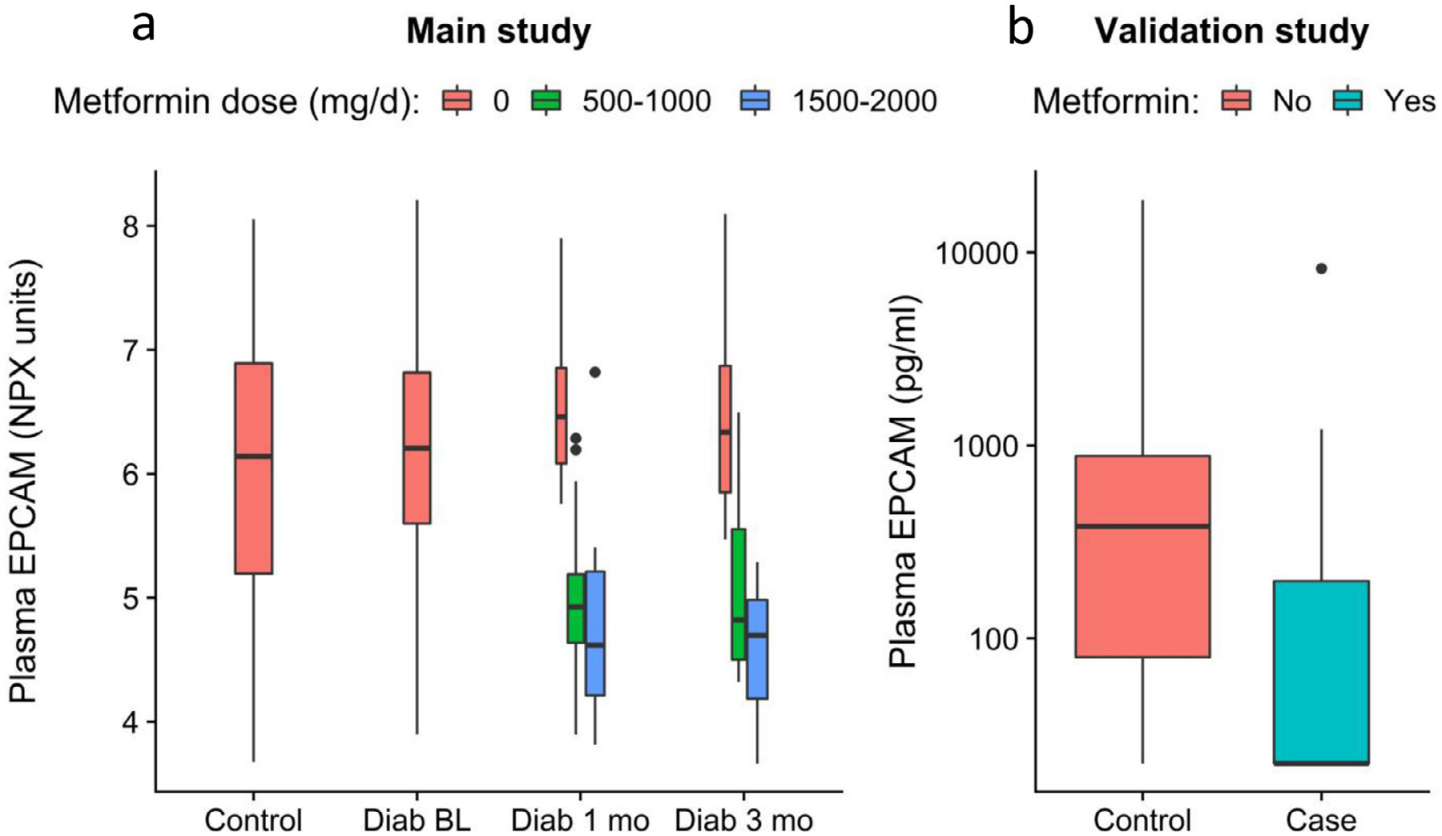
(a) Boxplots showing EPCAM levels in relation to metformin medication in the main study. Boxes represent median and the first and third quartiles of the data. (b) To validate the finding, plasma EPCAM was measured with ELISA in a separate cohort of 22 metformin-treated subjects and 44 non-treated controls that were matched according to age, sex, BMI and HbA1c. The metformin-treated subjects had significantly lower EPCAM levels than the controls (*p*-value=0.001 [Mann-Whitney U test, 1-sided], *n* = 66).

Due to the apparently large importance of metformin for the proteomic shift during treatment, we performed a validation study on EPCAM which was the protein that showed the strongest association with metformin in our data (Fig. 6a). From a separate population study we identified 22 metformin-treated subjects (mean ± SD; age 63.9 ± 4.0 years, BMI 30.4 ± 3.7 kg/m^2^, fasting glucose 6.9 ± 0.9 mmol/L) and control group of 44 subjects without metformin, matched based on age, sex, BMI and fasting glucose concentrations (mean ± SD; age 62.7 ± 4.5 years, BMI 29.6 ± 4.4 kg/m^2^, fasting glucose 6.8 ± 0.9 mmol/L). The proportion of men was 59% in both groups. The validation study confirmed the hypothesis that metformin medication is associated with reduced plasma EPCAM levels (p-value=0.001 [Mann–Whitney U test, 1-sided], Fig. 6b).

## 4 Discussion

Results from the present study show that subjects with screening detected early type 2 diabetes, void of classic diabetes symptoms, display wide-ranging alterations in the plasma proteome as compared to non-diabetic controls, to the extent that diabetes status can be predicted with high accuracy from the protein signature. These findings support the notion that broad biochemical alterations are present already at the onset of type 2 diabetes and that protein profiling could deliver individualized health assessments of cardiometabolic diseases.

Our study represents the most comprehensive PEA proteomics study of type 2 diabetes so far, measuring 974 unique proteins at multiple time points, revealing several plasma proteins not previously associated with diabetes. One example is ADGRG1 which is the most abundant G protein-coupled receptor in human pancreatic islets and plays an important role in pancreatic β-cell function [22], but has not previously been reported to be a circulating biomarker of diabetes. Another example is IGSF3 which is a little studied member of the immunoglobulin superfamily of proteins that appears to be completely unknown in the context of diabetes and cardiometabolic diseases. Although the mechanism that links IGSF3 to diabetes is unclear, there were several observations that makes IGSF3 an interesting candidate to study further, including strong correlations with insulin and liver fat, as well as the responsiveness to diabetes treatment. These examples, together with several other proteins that have not been described previously to associate with diabetes (e.g. HNMT, SIT1, RTN4R, CDCP1, SIGLEC10, IFNLR1 and VSIG4) expand the knowledge about the biochemical manifestations of type 2 diabetes and provides a resource for new candidate biomarkers in this disease area. This study also confirms several previously published associations between key proteins and prevalent diabetes and/or diabetes progression, including PON3, HGF, CTSD, IL1RA, SIGLEC7, LPL, IL6, FGF21, ERBB2, ALDH1A1, GAL4, ADM and FABP4 [5,[23], [24], [25], [26], [27], [28], [29], [30], [31], [32]].

Cardiovascular disease (CVD) is the primary cause of morbidity and mortality in people with diabetes, and already at this early stage of the disease the diabetes subjects displayed alterations in a range of CVD-associated proteins. Proteins implicated in the atherosclerotic process and suggested as potential blood-borne biomarkers of CVD include HGF, CTSD, IL6, TNFR1, IL1RA, FABP4, FGF21, and LPL [[24], [25], [26], [27],[32], [33], [34]]. Notably, the protein most predictive of diabetes status in the random forest model was NOS3 (also known as endothelial NOS) which is known to play a key role in CVD-protection via the generation of the vasodilator nitric oxide in blood vessels [35]. To our knowledge, there are no previous studies showing that diabetes is associated with elevated circulating NOS3. Our findings encourage future studies that evaluate integrated proteomics approaches to improve CVD risk stratification in diabetes subjects.

The proteomic variance that separated diabetes subjects from controls reflected key metabolic syndrome features including insulin resistance, hyperglycemia, fatty liver and adiposity. The importance of fatty liver was particularly striking among the top 30 diabetes-associated proteins (e.g. HGF, IGSF3, IL1RA, ALDH1A1, HNMT, ERBB2 and CDCP1). NAFLD is an important risk factor for liver disease as well as CVD [36], yet often goes undetected in the clinical routine due to the limited sensitivity of current liver function tests [37], and therefore improved biomarkers are needed. Our observation that NAFLD is a strong driver of plasma protein patterns is in line with two recent studies suggesting that protein profiling could potentially serve as a biomarker for NAFLD-screening [7,38].

The large majority of plasma proteins are stable over time in humans while healthy, and deviations from the individual´s trajectory could serve as a comprehensive indicator of changes in the health state [11]. A previous study in insulin resistant subjects indicated that the proteome is sensitive to periods of weight gain and loss [39], however plasma protein changes during improvements in glucose control are not well studied, nor is the potential influence of diabetes medication on the proteome. To better understand the dynamics of protein signatures in diabetes, it was therefore of key interest to investigate if metabolic improvement is reflected in the overall protein profile. Our data showed that during treatment, the overall proteome in the diabetes group shifted significantly towards the control group. This indicates that diabetes-associated protein patterns are responsive to treatment and hence might serve as a tool to elucidate the systemic effects of diabetes treatments in a broad and data-driven manner. This notion was further supported by our findings that metformin medication overshadowed the importance of glucose control for the overall proteome, and that the proteins that changed the most during treatment correlated strongly with metformin medication. This is interesting since the mechanisms by which metformin regulates blood glucose are only partly understood [40], and the data provided here might provide important clues to its pharmacological effects. Among the top metformin-associated proteins in our study was GDF15, which was initially discovered to be metformin-associated from screening 237 serum biomarkers [41], followed by further studies showing that GDF15 mediates the effect of metformin on body weight [42,43]. Our findings confirm that metformin treatment is associated with GDF15 levels, but also clarifies that diabetes is associated with increased GDF15 levels even in the absence of metformin. The protein most strongly associated with metformin in our data was EPCAM, a protein that is implicated in cancer pathophysiology and suggested as a circulating cancer biomarker [44]. This finding was intriguing in view of the growing body of evidence that metformin may prevent cancer [45] by mechanisms that are poorly understood. Some evidence that metformin reduces EPCAM expression exists from *in vitro* studies of cancer cells [46], but to our knowledge it has not previously been shown in humans that circulating EPCAM is associated with metformin. Whether EPCAM mediates any of the metabolic effects of metformin remains to be investigated.

One limitation of this study is the relatively small sample size. Even so, the corrected p-values for the associations discussed here were very low and the validity of our group-wise comparison is supported by the large overlap with the results in a recent cross-sectional proteomics study that searched for diabetes-associated proteins based on three of the 11 PEA panels used in the present study [5]. Novel findings should be verified in other cohorts to test external validity, given that our study population consists of middle-aged subjects of mainly European descent. Another limitation is that it is observational and hence cause-effect relationships cannot be inferred. The key strengths of this study are that it captures the very early phase of type 2 diabetes, that the group-wise comparisons are not confounded by diabetes treatment, the detailed phenotyping that enabled us to link protein signatures to various cardiometabolic features, and the longitudinal aspect showing the dynamics of protein signatures during treatment.

In conclusion, a broad range of blood-borne proteins are altered already at the very early stage of screening-detected type 2 diabetes, reflecting key metabolic syndrome features such as insulin resistance, fatty liver, hyperglycemia and adiposity. The comprehensive protein analyses revealed previously unknown associations with diabetes, as well as confirming previously published associations, thus contributing to our knowledge about the biochemical manifestations of diabetes. The overall proteomic alteration observed at baseline was significantly attenuated during metabolic improvement and appeared to be modified by metformin medication independent of metabolic effects. The results suggest that comprehensive protein profiling may serve as a useful tool for metabolic phenotyping and to elucidate the biological effects of diabetes treatment.

## Declaration of Competing Interest

The authors declare no conflicts of interest.

## Funding sources

This work was supported by the Swedish Heart-Lung Foundation (#20180324), the 10.13039/501100001862Swedish Research Council (2019-01140), the Erling Persson Foundation, the Knut and Alice Wallenberg Foundation, and the Swedish state under the agreement between the Swedish government and the county councils (ALFGBG-929989, ALFGBG-718851).

## Data sharing

The participant-level datasets used for this report have been deposited with the Swedish National Data Service (www.snd.gu.se, a data repository certified by Core Trust Seal). The dataset can be made available for validation purposes by contacting snd@snd.gu.se. Data access will be evaluated according to Swedish legislation. Data access for research related questions can be made available upon reasonable request by contacting the corresponding author.

## Appendix Supplementary materials

Image, application 1
