# Supplementary material for: Longitudinal plasma protein profiling of newly diagnosed type 2 diabetes
Source: eBioMedicine. 2020 Dec 3;63:103147. doi: 10.1016/j.ebiom.2020.103147 (PMC7718461; doi:10.1016/j.ebiom.2020.103147)
Supplement: Supplementary file 1 [file mmc1.docx]

**SUPPLEMENTAL DATA**

**Supplemental Table S1**: List of top 30 proteins showing significant difference between diabetes and control groups [Mann-Whitney U test, FDR-adjusted]. The proteins are ordered according to p-value (lowest to highest). Linear regression model p-values for the effect of diabetes status while adjusting for: ^a^ age and gender and ^b^ age, gender and BMI are also presented.

| **Protein** | **Log2 fold change** | **P-value** | **Linreg p-value^a^** | **Linreg p-value^b^** | **Implication in cardiometabolic diseases** |
| --- | --- | --- | --- | --- | --- |
| PON3 (Paraoxonase 3) | -0.197 | 1.03E-09 | 2.88E-14 | 8.79E-07 | Binds to HDL particles and inhibits the oxidative modification of LDL, protective properties against atherogenesis and adiposity in animal studies (PMID: 27771368). Type 2 diabetes is associated with reduced circulating concentration of PON3 (PMID: 31446444). |
| HGF (Hepatocyte growth factor) | 0.106 | 1.03E-09 | 1.13E-15 | 2.38E-08 | Growth factor for a broad spectrum of tissues and cell types. High circulating HGF concentration predicts future diabetes (PMID: 26892517) and progression of atherosclerosis (PMID: 29609131). |
| NOS3 (Nitric oxide synthase 3) | 0.494 | 3.55E-09 | 2.71E-16 | 3.52E-09 | Generates the vasodilator nitric oxide (NO) in blood vessels, which has vasoprotective and anti-atherosclerotic effects by a variety of mechanisms (PMID: 21890489). |
| CTSD (Cathepsin D) | 0.316 | 3.31E-07 | 1.89E-11 | 5.76E-07 | Induces hydrolytic modification of LDL, contributing to the accumulation of modified LDL in the arterial intima (PMID: 12750117). High CTSD concentration is associated with diabetes and increased risk of coronary events (PMID: 26848396). |
| IGSF3 (Immunoglobulin superfamily member 3) | 0.143 | 5.2E-07 | 1.35E-09 | 4.49E-05 | No clear relation with cardiometabolic diseases. |
| IL1RA (Interleukin 1 receptor antagonist) | 0.254 | 6.82E-07 | 1.10E-12 | 4.61E-05 | Natural inhibitor of the pro-inflammatory effect of IL1β. Circulating IL1RA concentration is increased in type 2 diabetes, and associated with risk of CVD in a meta-analysis of 6 population-based cohorts (PMID: 28428221). |
| SULT2A1 (Sulfotransferase family 2A member 1) | 3.925 | 8.22E-07 | 3.96E-10 | 4.23E-05 | Catalyzes the sulfonation of steroids and bile acids in the liver and adrenal glands. No clear relation with cardiometabolic diseases. |
| SIGLEC7 (Sialic acid binding Ig like lectin 7) | 0.117 | 8.22E-07 | 1.43E-10 | 8.68E-06 | Over-expression of SIGLEC7 in diabetic islets reduced cytokines, prevented β-cell dysfunction and apoptosis and reduced recruiting of migrating monocytes (PMID: 28378743). Circulating concentration of SIGLEC7 is increased in diabetes (PMID: 31446444). |
| ADGRG1 (Adhesion G protein-coupled receptor G1) | 0.724 | 1.11E-06 | 1.21E-11 | 9.87E-07 | The most abundant G protein-coupled receptor in human pancreatic islets and plays an important role in pancreatic β-cell function (PMID: 27636017). |
| CPM (Carboxypeptidase M) | 0.035 | 1.19E-06 | 1.99E-09 | 5.72E-04 | Key enzyme in the generation of B1 receptor agonists which are associated with vascular inﬂammation, insulin resistance, diabetic complications, and activation of the inducible form of NOS (iNOS) (PMID: 28824433) |
| HNMT (Histamine N-methyltransferase) | 0.095 | 1.86E-06 | 2.11E-09 | 1.26E-04 | No clear relation with cardiometabolic diseases. |
| SIT1 (Signaling threshold regulating transmembrane adaptor 1) | 0.233 | 2.25E-06 | 7.17E-09 | 4.85E-04 | No clear relation with cardiometabolic diseases. |
| LPL (Lipoprotein lipase) | -0.064 | 2.29E-06 | 5.56E-10 | 2.50E-05 | Key enzyme in triglyceride metabolism, hydrolyzes fatty acids from triglyceride-rich lipoproteins and regulates their distribution to peripheral tissues. Reduced levels of serum LPL is associated with diabetes and increased risk for future CAD (PMID: 16373616). |
| RTN4R (Reticulon 4 receptor) | 0.248 | 2.29E-06 | 1.41E-09 | 3.13E-04 | No clear relation with cardiometabolic diseases. |
| IL6 (Interleukin-6) | 0.416 | 2.75E-06 | 5.00E-11 | 4.56E-05 | Cytokine with a wide variety of biological functions, potent inducer of the acute phase response. Circulating concentration of IL6 predicts macrovascular events in individuals with diabetes (PMID: 24222348). |
| TNFR1 (Tumor necrosis factor receptor 1) | 0.069 | 3.06E-06 | 5.87E-09 | 4.06E-05 | Circulating concentration of TNFR1 predicts CV events in individuals with diabetes (PMID: 29796748). |
| FGF21 (Fibroblast growth factor 21) | 0.227 | 3.1E-06 | 8.27E-10 | 5.54E-05 | Peptide hormone that regulates energy homeostasis in multiple target organs. Circulating FGF21 concentration is associated with diabetes, subclinical atherosclerosis and cardiovascular events (PMID: 28012645). |
| ALDH1A1 (Aldehyde dehydrogenase 1 family member A1) | 0.373 | 3.14E-06 | 1.80E-09 | 1.76E-04 | Converts retinaldehyde to retinoic acid. ALDH1A1-deficient mice display decreased fasting glucose levels and hepatic gluconeogenesis (PMID: 22555438). Type 2 diabetes is associated with increased circulating concentration of ALDH1A1 (PMID: 31446444). |
| CDCP1 (CUB domain containing protein 1) | 0.251 | 3.14E-06 | 1.03E-10 | 9.53E-06 | No clear relation with cardiometabolic diseases. |
| GAL4 (Galectin 4) | 0.188 | 4.12E-06 | 1.98E-09 | 1.76E-07 | Galectin that binds lactose and a related range of sugars. Increased circulating concentration of GAL4 is associated with incident diabetes (PMID: 30670722). |
| THBS2 (Thrombospondin-2) | 0.041 | 4.12E-06 | 3.77E-09 | 4.18E-06 | Involved in cell-matrix interactions. Hyperglycemia-induced THBS2 expression contributes to impaired healing in diabetes (PMID: 31391172). |
| ANGPTL4 (Angiopoietin-related protein 4) | 0.142 | 4.12E-06 | 5.35E-09 | 2.22E-05 | Mediates inactivation of the lipoprotein lipase LPL. Genetic inactivation of ANGPTL4 is associated with improved glucose homeostasis and reduced risk of diabetes (PMID: 29899519). |
| ERBB2 (Erb-b2 receptor tyrosine kinase 2) | 0.042 | 4.12E-06 | 3.08E-09 | 1.78E-04 | Protein tyrosine kinase that is part of several cell surface receptor complexes. Circulating ERBB2 concentration is associated with hyperglycemia, insulin resistance and incident diabetes (PMID: 31201260). |
| IL18R1 (Interleukin 18 receptor 1) | 0.071 | 4.28E-06 | 1.74E-08 | 1.36E-04 | Contributes to IL18-induced cytokine production. Elevated circulating IL18 is associated with increased risk of type 2 diabetes (PMID: 16186395), however the association with IL18R1 is unclear. |
| SIGLEC10 (Sialic acid binding Ig like lectin 10) | 0.113 | 4.28E-06 | 3.62E-09 | 7.79E-05 | No clear relation with cardiometabolic diseases. |
| ADM (Adrenomedullin) | 0.066 | 6.03E-06 | 8.16E-10 | 7.70E-04 | Hormone with vasodilatatory and hypotensive effects, inhibits ACTH secretion. Circulating ADM concentrations are increased in diabetes (PMID: 24936257). ADM suppresses insulin synthesis and secretion by pancreatic β-cells in vitro (PMID: 30383252). |
| FABP4 (Fatty acid binding protein 4) | 0.169 | 7.54E-06 | 2.73E-12 | 3.50E-04 | Transports long chain fatty acids and retinoic acid in adipocytes. Increased levels of FABP4 is associated with diabetes and cardiovascular events (PMID: 30726793). |
| IFNLR1 (Interferon lambda receptor 1) | 0.075 | 1.16E-05 | 0.029 | 0.097 | No clear relation with cardiometabolic diseases. |
| VSIG4 (V-set and immunoglobulin domains-containing protein 4) | 0.088 | 1.35E-05 | 2.12E-08 | 9.74E-05 | No clear relation with cardiometabolic diseases. |
| APOM (Apolipoprotein M) | -0.055 | 1.57E-05 | 2.19E-07 | 0.0013 | A minor HDL apolipoprotein. Low circulating APOM is associated with atherosclerosis progression in rodents and with increased mortality in humans with diabetes (PMID: 31133557). |

**Supplemental Table S2**: List of all proteins showing significant differences between diabetes and control group, with log2 fold change and FDR-adjusted p-values for the group-wise comparisons (Mann-Whitney U test). The proteins are ordered according to p-value for group difference (lowest to highest).

| **Protein name** | **Log2 fold change** | **P-value** |
| --- | --- | --- |
| PON3 | -0.19734 | 1.03E-09 |
| HGF | 0.106255 | 1.03E-09 |
| NOS3 | 0.494377 | 3.55E-09 |
| CTSD | 0.315685 | 3.31E-07 |
| IGSF3 | 0.143275 | 5.2E-07 |
| IL-1ra | 0.253506 | 6.82E-07 |
| SULT2A1 | 3.924999 | 8.22E-07 |
| SIGLEC7 | 0.117147 | 8.22E-07 |
| ADGRG1 | 0.724177 | 1.11E-06 |
| CPM | 0.035337 | 1.19E-06 |
| HNMT | 0.094979 | 1.86E-06 |
| SIT1 | 0.232886 | 2.25E-06 |
| LPL | -0.0641 | 2.29E-06 |
| RTN4R | 0.24803 | 2.29E-06 |
| IL6 | 0.416271 | 2.75E-06 |
| TNF-R1 | 0.06869 | 3.06E-06 |
| FGF-21 | 0.226913 | 3.1E-06 |
| ALDH1A1 | 0.373329 | 3.14E-06 |
| CDCP1 | 0.251276 | 3.14E-06 |
| Gal-4 | 0.188026 | 4.12E-06 |
| THBS2 | 0.040565 | 4.12E-06 |
| ANGPTL4 | 0.14185 | 4.12E-06 |
| ERBB2 | 0.041612 | 4.12E-06 |
| IL-18R1 | 0.071079 | 4.28E-06 |
| SIGLEC10 | 0.112837 | 4.28E-06 |
| ADM | 0.065986 | 6.03E-06 |
| FABP4 | 0.168732 | 7.54E-06 |
| IFNLR1 | 0.07504 | 1.16E-05 |
| VSIG4 | 0.088273 | 1.35E-05 |
| APOM | -0.05491 | 1.57E-05 |
| CTSO | 0.14748 | 2.05E-05 |
| FURIN | 0.053282 | 2.05E-05 |
| TNFRSF11A | 0.082152 | 2.05E-05 |
| PXN | 0.163004 | 2.18E-05 |
| SERPINA7 | 0.073309 | 2.2E-05 |
| CDH2 | 0.130137 | 2.2E-05 |
| Gal-9 | 0.037967 | 2.67E-05 |
| HAOX1 | 0.409437 | 2.67E-05 |
| NFATC3 | 0.472108 | 3.03E-05 |
| GDF-15 | 0.121557 | 3.5E-05 |
| SPON2 | 0.018492 | 3.5E-05 |
| CHI3L1 | 0.319564 | 3.5E-05 |
| ICAM1 | 0.064176 | 3.5E-05 |
| CCL19 | 0.092999 | 3.5E-05 |
| PAI | 0.13621 | 4.17E-05 |
| TNFRSF6B | 0.113339 | 4.55E-05 |
| KYNU | 0.097586 | 4.77E-05 |
| SELE | 0.062781 | 4.77E-05 |
| OSM | 0.268916 | 4.77E-05 |
| FAS | 0.057162 | 4.77E-05 |
| FBP1 | 0.299272 | 4.77E-05 |
| GUSB | 0.123562 | 4.78E-05 |
| CTSZ | 0.090126 | 5.03E-05 |
| LILRA5 | 0.090026 | 5.03E-05 |
| PRSS8 | 0.043493 | 5.03E-05 |
| IGFBP-2 | -0.09889 | 5.27E-05 |
| FGF-23 | 0.160345 | 5.88E-05 |
| CD163 | 0.074017 | 6.85E-05 |
| CSF-1 | 0.025769 | 7.48E-05 |
| GCG | 0.275308 | 9.45E-05 |
| TRAIL-R2 | 0.067376 | 0.000101 |
| MVK | 0.199794 | 0.000115 |
| NOMO1 | 0.078518 | 0.000118 |
| TIMP1 | 0.059466 | 0.000122 |
| MSR1 | 0.073788 | 0.000134 |
| HAVCR2 | 0.109824 | 0.00015 |
| INHBC | 0.11482 | 0.000154 |
| SIGLEC1 | 0.08274 | 0.000164 |
| THOP1 | 0.066136 | 0.000165 |
| CCL15 | 0.071685 | 0.000166 |
| DDAH1 | 0.243348 | 0.000166 |
| CALCA | 0.142856 | 0.000166 |
| CD28 | 0.147873 | 0.000166 |
| MRC2 | 0.125361 | 0.000192 |
| BCAN | -0.07456 | 0.000197 |
| CA5A | 0.320327 | 0.000215 |
| CCL3 | 0.092415 | 0.000225 |
| TNFRSF10A | 0.134025 | 0.000231 |
| IL8 | 0.104646 | 0.000262 |
| U-PAR | 0.07471 | 0.000269 |
| IL16 | 0.061556 | 0.00027 |
| ENPP7 | 0.23716 | 0.0003 |
| CD4 | 0.049025 | 0.000308 |
| ACE2 | 0.181178 | 0.000323 |
| TWEAK | -0.03916 | 0.000332 |
| CCL21 | 0.149215 | 0.000334 |
| TNF-R2 | 0.071157 | 0.000371 |
| MMP12 | 0.094466 | 0.000404 |
| CCL16 | 0.082243 | 0.000456 |
| 5'-NT | 0.056845 | 0.000468 |
| TREM1 | 0 | 0.000481 |
| TNFSF13B | 0.051413 | 0.000513 |
| KIM1 | 0.081353 | 0.000557 |
| CD83 | 0.087855 | 0.000562 |
| CCL20 | 0.130145 | 0.000566 |
| t-PA | 0.096435 | 0.000627 |
| TMPRSS5 | -0.09141 | 0.000644 |
| NINJ1 | 0.081071 | 0.000686 |
| APLP1 | -0.11026 | 0.000705 |
| CES1 | 0.307403 | 0.000711 |
| SEZ6L | -0.06288 | 0.000717 |
| LILRB2 | 0.102251 | 0.000723 |
| N2DL-2 | 0.085399 | 0.000729 |
| IL-1RT2 | 0.060508 | 0.000791 |
| NCF2 | 0.106262 | 0.000791 |
| SEMA3F | 0.078301 | 0.000791 |
| MCP-3 | 0.176076 | 0.000798 |
| G-CSF | 0.178927 | 0.000805 |
| OMD | -0.11658 | 0.000881 |
| PGF | 0.024263 | 0.000914 |
| PRCP | 0.157027 | 0.000922 |
| GALNT2 | 0.040653 | 0.001055 |
| SCF | -0.04332 | 0.001055 |
| WNT9A | -0.08198 | 0.001055 |
| C2 | 0.033306 | 0.001095 |
| CST3 | 0.039975 | 0.001095 |
| TR-AP | 0.101299 | 0.001095 |
| VEGFA | 0.03102 | 0.001095 |
| MMP7 | 0.028867 | 0.001145 |
| SSC4D | 0.384784 | 0.001219 |
| TPP1 | 0.087445 | 0.001467 |
| CA6 | -0.27264 | 0.00148 |
| C1QTNF1 | 0.11047 | 0.00163 |
| IL-10RB | 0.04361 | 0.001733 |
| CA14 | -0.07255 | 0.00181 |
| PARP-1 | 0.170282 | 0.001859 |
| PODXL2 | -0.04259 | 0.001876 |
| CTSS | 0.025584 | 0.002031 |
| HSD11B1 | -0.11649 | 0.002031 |
| SCARB2 | 0.076811 | 0.002031 |
| LEP | 0.140713 | 0.002121 |
| CLEC7A | 0.140473 | 0.002141 |
| LAIR1 | 0.087014 | 0.002198 |
| ACAN | -0.08079 | 0.002219 |
| MMP-9 | 0.114949 | 0.00224 |
| EGFL7 | 0.052875 | 0.002283 |
| PON2 | 0.180947 | 0.002283 |
| SLAMF1 | 0.288029 | 0.002344 |
| LDLreceptor | 0.114767 | 0.002407 |
| EDAR | -0.17025 | 0.00243 |
| IDUA | 0.064992 | 0.002454 |
| CLUL1 | -0.12304 | 0.002605 |
| CRH | -0.25465 | 0.002631 |
| EGLN1 | 0.17109 | 0.002746 |
| MOG | -0.07917 | 0.003062 |
| STC1 | 0.061279 | 0.003143 |
| FCGR2A | 0.120979 | 0.003173 |
| SIGLEC6 | 0.060013 | 0.003311 |
| CD48 | 0.034355 | 0.003569 |
| LILRB1 | 0.189635 | 0.003603 |
| SEMA7A | 0.058067 | 0.003819 |
| CLM-6 | 0.034203 | 0.003825 |
| CD59 | 0.079541 | 0.003957 |
| ALDH3A1 | 0.639276 | 0.004166 |
| IGF2R | 0.038574 | 0.004166 |
| COLEC12 | 0.050712 | 0.004274 |
| IL2-RA | 0.11172 | 0.004316 |
| ITGA5 | 0.070983 | 0.004358 |
| RARRES2 | 0.018786 | 0.004616 |
| AGRP | 0.093133 | 0.004631 |
| CD5 | 0.055423 | 0.004631 |
| FETUB | 0.173961 | 0.004648 |
| REN | 0.099986 | 0.004648 |
| CLEC4G | 0.078891 | 0.004769 |
| SERPINA5 | 0.032543 | 0.00497 |
| PRELP | 0.014656 | 0.005019 |
| LILRB4 | 0.094172 | 0.005199 |
| ZBTB17 | 0.11934 | 0.005199 |
| CLEC6A | 0.175699 | 0.005333 |
| NCAN | -0.03271 | 0.005386 |
| CYR61 | 0.0654 | 0.005439 |
| TGFR-2 | 0.037621 | 0.005756 |
| TGF-alpha | 0.097352 | 0.005996 |
| S100A11 | 0.052222 | 0.006055 |
| CCL4 | 0.063809 | 0.006504 |
| IL18 | 0.054347 | 0.006735 |
| NEP | 0.194039 | 0.006735 |
| ADGRE2 | 0.083041 | 0.006763 |
| WFIKKN2 | -0.06054 | 0.006763 |
| PCDH17 | 0.082551 | 0.00715 |
| GHRL | -0.22884 | 0.00722 |
| IQGAP2 | 0.165951 | 0.007631 |
| NTRK3 | -0.02399 | 0.007943 |
| CXCL13 | 0.054846 | 0.00802 |
| GALNT10 | 0.129456 | 0.008054 |
| IL12RB1 | 0.124851 | 0.008054 |
| CRTAC1 | -0.18958 | 0.008213 |
| GH | -0.16226 | 0.008213 |
| FSTL3 | 0.078735 | 0.008249 |
| ST2 | 0.102945 | 0.008249 |
| SERPINB8 | 0.096263 | 0.008413 |
| CADM3 | -0.11107 | 0.008413 |
| MCP-1 | 0.050799 | 0.008495 |
| ADAM8 | 0.058368 | 0.008664 |
| NADK | 0.097053 | 0.008664 |
| DLL1 | 0.026009 | 0.009426 |
| PLXNB2 | 0.1426 | 0.009518 |
| EPO | 0.304204 | 0.010047 |
| CXCL10 | 0.055134 | 0.010296 |
| IGFBPL1 | 0.124926 | 0.011024 |
| N-CDase | 0.114218 | 0.011544 |
| CLSTN3 | 0.097244 | 0.012027 |
| MATN3 | -0.02703 | 0.012027 |
| CCL18 | 0.063916 | 0.01232 |
| IL-15RA | 0.088997 | 0.012439 |
| APEX1 | 0.209386 | 0.012498 |
| CD300LG | -0.06018 | 0.012498 |
| IGFBP-7 | 0.040896 | 0.012618 |
| GCP5 | -0.10144 | 0.013112 |
| MFGE8 | 0.071269 | 0.013238 |
| CES2 | 0.128587 | 0.013494 |
| VSIG2 | 0.099389 | 0.013494 |
| PAMR1 | 0.046623 | 0.013624 |
| IL-18BP | 0.045109 | 0.013953 |
| WFDC2 | 0.024366 | 0.014088 |
| EFNA4 | 0.064249 | 0.014158 |
| ENTPD2 | 0.119353 | 0.014158 |
| FGF-BP1 | -0.04598 | 0.014433 |
| TGFBR3 | -0.11203 | 0.014433 |
| PSGL-1 | 0.025858 | 0.015204 |
| LGMN | 0.055469 | 0.01535 |
| MASP1 | 0.076724 | 0.015497 |
| LXN | 0.08145 | 0.016549 |
| AGR3 | 0.33018 | 0.017421 |
| AXL | 0.025758 | 0.017586 |
| RET | 0.058025 | 0.018001 |
| LOX-1 | 0.053997 | 0.018344 |
| TIMD4 | 0.088823 | 0.018344 |
| SDC4 | -0.09646 | 0.018518 |
| MPO | 0.082167 | 0.018953 |
| SMPD1 | 0.058094 | 0.019133 |
| PLXNB3 | -0.05752 | 0.019314 |
| CD38 | 0.052395 | 0.019413 |
| FUT3/FUT5 | 0.107194 | 0.019413 |
| THY1 | 0.021175 | 0.019598 |
| EFEMP1 | 0.066194 | 0.019887 |
| ITGA6 | -0.06401 | 0.019887 |
| ITGAV | -0.02867 | 0.019887 |
| VEGFD | -0.03316 | 0.020352 |
| CEACAM5 | 0.308045 | 0.020545 |
| PLC | 0.022064 | 0.02074 |
| ESM-1 | -0.02758 | 0.020937 |
| TNFRSF13B | 0.025324 | 0.022613 |
| TMPRSS15 | 0.241687 | 0.023764 |
| CLEC4D | 0.195875 | 0.024536 |
| FCRL1 | 0.055095 | 0.024536 |
| ANGPT2 | 0.118091 | 0.024665 |
| SKAP1 | 0.061428 | 0.024665 |
| CHL1 | -0.06061 | 0.025228 |
| XCL1 | 0.043411 | 0.025803 |
| AREG | 0.098129 | 0.025837 |
| NMNAT1 | 0.123987 | 0.025837 |
| OMG | -0.1494 | 0.025837 |
| CD74 | 0.149014 | 0.027131 |
| LRP11 | 0.048422 | 0.028745 |
| RELT | 0.05051 | 0.028745 |
| PGLYRP1 | 0.033901 | 0.02901 |
| GAS6 | 0.059419 | 0.030326 |
| TYRO3 | 0.064843 | 0.030326 |
| GAL | -0.0801 | 0.032106 |
| NAAA | -0.07004 | 0.032106 |
| GLB1 | 0.081335 | 0.032275 |
| MIC-A/B | 0.13493 | 0.032275 |
| CD97 | 0.056643 | 0.032447 |
| LILRB5 | 0.126114 | 0.032447 |
| TNFRSF4 | 0.063942 | 0.032743 |
| CXADR | 0.151938 | 0.033344 |
| IL5 | -0.71542 | 0.033344 |
| SCARA5 | 0.013907 | 0.034081 |
| ADGRG2 | -0.05745 | 0.034832 |
| MARCO | 0.020316 | 0.035149 |
| MET | -0.04731 | 0.03638 |
| Beta-NGF | 0.108363 | 0.037511 |
| F7 | 0.039895 | 0.037511 |
| SKR3 | 0.021838 | 0.038328 |
| AMBP | 0.010027 | 0.038673 |
| AZU1 | 0.105095 | 0.03902 |
| SHPS-1 | 0.074637 | 0.03937 |
| CFC1 | 0.17435 | 0.039724 |
| F11 | 0.020573 | 0.04008 |
| ITGA11 | -0.07255 | 0.041979 |
| CD79B | 0.067475 | 0.042203 |
| PVR | 0.027327 | 0.042203 |
| CLM-1 | 0.059125 | 0.042958 |
| RGMA | -0.01839 | 0.042958 |
| AIFM1 | 0.145571 | 0.043341 |
| GDF-2 | -0.02771 | 0.044269 |
| COL18A1 | 0.05215 | 0.045214 |
| DDR1 | 0.020551 | 0.046177 |
| LAMP3 | 0.070564 | 0.046996 |
| NPDC1 | 0.029982 | 0.046996 |
| IL33 | 0.126458 | 0.047992 |
| CEACAM8 | 0.067183 | 0.048414 |

**Supplemental Table S3.** Clinical variables at baseline and after 1 and 3 months of treatment in the diabetes group. Values are median (interquartile range). The symbol * denotes p<0.001 and † denotes p<0.05 for change from baseline [paired Wilcoxon signed rank test].

| **Abbreviation** | **Clinical variable** | **Baseline (n=52)** | **1 month (n=48)** | **3 months (n=51)** |
| --- | --- | --- | --- | --- |
| SedentaryTime | Sedentary time (hours) | 8.0 (5.0) | 7.0 (4.5) | 7.5 (6.0) |
| Weight | Body weight (kg) | 94.8 (29.9) | 91.5 (31.0) * | 90.7 (31.7) * |
| BMI | Body mass index (kg/m^2^) | 31.9 (9.9) | 31.8 (9.6) * | 30.8 (9.9) * |
| Waist | Waist circumference (cm) | 108.5 (25.3) | 107.0 (26.3) † | 104.0 (26.5) * |
| Hip | Hip circumference (cm) | 109.5 (17.0) | 109.5 (18.0) | 109.0 (16.5) * |
| Bodyfat | Body fat content (%) | 32.4 (13.2) | 32.0 (14.0) * | 32.2 (14.6) * |
| SBP | Systolic blood pressure (mmHg) | 131.5 (23.0) | 128.0 (18.5) † | 127.0 (15.0) † |
| DBP | Diastolic blood pressure (mmHg) | 86.0 (13.5) | 84.0 (14.0) † | 83.0 (15.0) † |
| Gluc | Glucose (mmol/L) | 7.5 (1.6) | 6.8 (1.1) * | 6.7 (1.2) * |
| HbA1c | Hemoglobin A1c (mmol/mol) | 43.0 (7.5) | 40.5 (6.0) * | 38.0 (6.0) * |
| Insulin | Insulin (mU/L) | 11.3 (11.4) | 11.0 (10.8) | 11.4 (9.9) |
| HOMA-IR | Homeostatic model assessment of insulin resistance | 3.9 (3.7) | 3.6 (3.8) | 3.9 (2.8) |
| C-peptide | C-peptide (nmol/L) | 0.96 (0.63) | 0.89 (0.56) | 0.90 (0.57) |
| LDL-C | Low density lipoprotein cholesterol (mmol/L) | 3.3 (1.0) | 3.0 (1.3) * | 2.7 (1.2) * |
| HDL-C | High density lipoprotein cholesterol (mmol/L) | 1.4 (0.5) | 1.4 (0.5) | 1.3 (0.5) † |
| TG | Triglycerides (mmol/L) | 1.5 (0.6) | 1.4 (0.7) | 1.2 (0.8) |
| ApoA1 | Apolipoprotein A1 (g/L) | 1.5 (0.3) | 1.5 (0.3) | 1.5 (0.3) † |
| ApoB | Apolipoprotein B (g/L) | 1.0 (0.3) | 1.0 (0.3) | 0.92 (0.3) * |
| ALAT | Alanine aminotransferase (µkat/L) | 0.55 (0.36) | 0.55 (0.33) | 0.47 (0.31) † |
| GGT | Gamma glutamyltransferase (µkat/L) | 0.62 (0.55) | 0.51 (0.49) * | 0.48 (0.52) † |
| eGFR | Estimated glomerular filtration rate (mL/min/1.73 m2) | 77.4 (13.0) | 77.4 (15.6) | 76.0 (16.5) |
| NT-proBNP | N-terminal pro b-type natriuretic peptide (NT-pro-BNP) (ng/L) | 43.5 (59.8) | 54.0 (72.5) | 62.0 (75.0) † |
| Urate | Urate (µmol/L) | 363.5 (114.3) | 366.0 (113.8) | 356.0 (93.5) |
| CRP | C-reactive protein, high sensitivity (mg/L) | 2.6 (3.6) | 2.2 (3.5) † | 2.0 (2.9) † |
| WBC | White blood cell count (x10*9/L) | 5.8 (2.3) | 5.9 (2.3) | 6.0 (2.15) |
| Hb | Hemoglobin (g/L) | 147.0 (14.3) | 146.5 (15.3) | 143.0 (14.0) † |
| RBC | Red blood cell count (x10*12/L) | 4.8 (0.4) | 4.7 (0.5) | 4.6 (0.5) |
| Platelets | Platelet count (x10*9/L) | 210.5 (68.8) | 207.0 (77.3) | 215.0 (71.0) |

**Supplemental Table S4**: List of proteins showing significant changes from baseline to 3 months of diabetes treatment in the diabetes group (Mann-Whitney U test, FDR-adjusted). Indicated are also p-values of the 1-month vs baseline and p-values of the association with metformin treatment [mixed model, FDR-adjusted]. The proteins are ordered according to p-value for treatment effect (lowest to highest). Standardized beta coefficients (indicating direction of effect) for possible metformin-and visit effect are also shown.

|  | **Diabetes treatment effect** | | | **Metformin effect** | **Standardized Beta coefficients** | |
| --- | --- | --- | --- | --- | --- | --- |
| **Protein** | **Log2 FC (3 months vs baseline)** | **P-value**  **(3 months vs baseline)** | **P-value**  **(1 month vs baseline)** | **P-value mixed model** | **Std Beta metformin** | **Std Beta visit** |
| REG4 | 0.087632 | 2.80E-06 | 3.0E-05 | **6.38E-05** | 0.261 | 0.059 |
| EPCAM | -0.29054 | 2.80E-06 | 1.0E-05 | **2.65E-09** | -0.665 | -0.022 |
| CPA2 | 0.087699 | 8.10E-06 | 1.1E-06 | **6.66E-04** | 0.283 | 0.071 |
| GDF15 | 0.113339 | 1.3E-05 | 1.2E-04 | **5.62E-08** | 0.33 | -0.023 |
| PCDH17 | -0.05341 | 2.6E-05 | 0.0016 | **2.60E-04** | -0.133 | 0.001 |
| COL1A1 | -0.08136 | 1.5E-04 | 0.679 | 0.885 | 0.018 | -0.083 |
| SELE | -0.02676 | 1.6E-04 | 0.0036 | 0.661 | -0.041 | -0.067 |
| SPINK1 | 0.118639 | 3.1E-04 | 0.00309 | **1.23E-04** | 0.233 | 0.006 |
| IGFBP2 | 0.045488 | 0.0018 | 0.0293 | 0.858 | 0.03 | 0.079 |
| RET | -0.04347 | 0.0018 | 0.00162 | 0.519 | -0.044 | -0.046 |
| SLITRK2 | -0.04207 | 0.0018 | 0.00359 | **0.013** | -0.094 | 0.003 |
| ITGB2 | -0.04666 | 0.0022 | 0.00814 | 0.167 | -0.069 | -0.034 |
| FCN2 | -0.03831 | 0.0024 | 0.0203 | 0.658 | -0.037 | -0.04 |
| IGSF3 | -0.06579 | 0.0024 | 0.00234 | **0.030** | -0.103 | -0.015 |
| CDH5 | -0.03795 | 0.0024 | 0.0335 | 0.554 | -0.042 | -0.023 |
| LRRN1 | -0.05495 | 0.0035 | 0.00248 | 0.077 | -0.076 | -0.013 |
| UNC5C | 0.04025 | 0.0038 | 0.0322 | 0.129 | 0.07 | 0.021 |
| SEMA7A | -0.03439 | 0.0044 | 0.031 | 0.578 | -0.034 | -0.042 |
| THBS4 | -0.08649 | 0.0056 | 0.00258 | **0.022** | -0.172 | -0.011 |
| IL18R1 | -0.025 | 0.0059 | 0.00164 | **0.013** | -0.097 | 0.001 |
| LDLR | -0.07439 | 0.0059 | 0.031 | 0.297 | -0.082 | -0.038 |
| TFPI | -0.02177 | 0.0071 | 0.0887 | 0.913 | -0.015 | -0.046 |
| C1QTNF1 | -0.07398 | 0.0072 | 0.00735 | 0.435 | -0.068 | -0.036 |
| LTBR | -0.03862 | 0.0076 | 0.0764 | 0.941 | -0.013 | -0.035 |
| UPA | -0.03342 | 0.0076 | 0.19 | 0.999 | -0.002 | -0.045 |
| MUC16 | -0.04099 | 0.0076 | 0.11 | 0.263 | -0.073 | -0.008 |
| ERBB2 | -0.01738 | 0.01 | 0.0203 | 0.540 | -0.03 | -0.026 |
| REG1A | 0.051847 | 0.01 | 0.0501 | **0.043** | 0.142 | 0.008 |
| LEP | -0.0467 | 0.016 | 0.0555 | 0.908 | -0.027 | -0.08 |
| CNTN1 | -0.03166 | 0.016 | 0.0791 | 0.416 | -0.047 | -0.012 |
| CTSH | -0.05761 | 0.017 | 0.12 | 0.222 | -0.056 | -0.008 |
| PTPRJ | 0.272708 | 0.017 | 0.546 | 0.947 | -0.05 | 0.274 |
| FAM3B | 0.060093 | 0.021 | 0.104 | 0.372 | 0.071 | 0.042 |
| AMIGO2 | -0.02551 | 0.021 | 0.0259 | **0.033** | -0.06 | 0.005 |
| ALCAM | -0.01832 | 0.021 | 0.344 | 0.685 | -0.026 | -0.022 |
| CDHR5 | -0.03971 | 0.021 | 0.19 | 0.796 | -0.024 | -0.032 |
| FAM3C | 0.034407 | 0.022 | 0.322 | 0.614 | 0.041 | 0.04 |
| MMP1 | 0.04532 | 0.022 | 0.104 | 0.341 | 0.142 | 0.046 |
| ADGRG1 | -0.06773 | 0.025 | 0.00309 | 0.424 | -0.057 | -0.019 |
| VSIG2 | 0.043362 | 0.028 | 0.847 | 0.971 | 0.009 | 0.047 |
| PAI | -0.0646 | 0.028 | 0.00102 | 0.511 | -0.093 | -0.055 |
| CDH2 | -0.0579 | 0.028 | 0.031 | 0.075 | -0.1 | -0.01 |
| TFF3 | 0.034564 | 0.028 | 0.013 | **0.022** | 0.146 | -0.047 |
| DLK1 | -0.03568 | 0.028 | 0.00359 | 0.344 | -0.069 | -0.017 |
| ICAM1 | -0.02729 | 0.028 | 0.102 | 0.297 | -0.069 | -0.009 |
| MK | 0.042642 | 0.031 | 0.185 | 0.631 | 0.048 | 0.05 |
| LRMP | 0.124786 | 0.032 | 0.496 | 0.897 | 0.066 | 0.139 |
| NTRK2 | -0.01776 | 0.033 | 0.000538 | 0.191 | -0.039 | -0.005 |
| HSP27 | 0.010546 | 0.033 | 0.116 | 0.971 | 0.006 | 0.031 |
| GUSB | -0.05062 | 0.037 | 0.0567 | 0.999 | 0 | -0.075 |
| IGF2R | -0.02627 | 0.037 | 0.0137 | 0.288 | -0.063 | -0.017 |
| ALDH1A1 | -0.13841 | 0.038 | 0.116 | 0.831 | -0.045 | -0.083 |
| NTproBNP | 0.15645 | 0.047 | 0.157 | 0.687 | -0.117 | 0.28 |
| AREG | 0.077658 | 0.048 | 0.344 | 0.894 | 0.024 | 0.048 |
| PIgR | 0.011774 | 0.048 | 0.189 | 0.091 | 0.035 | 0.001 |
| WAS | 0.201481 | 0.048 | 0.884 | 0.999 | -0.006 | 0.29 |
| CA5A | -0.10455 | 0.049 | 0.256 | 0.989 | 0.016 | -0.133 |

**Supplemental Figure S1:** A-C) Overall protein profiles for subjects in the diabetes group (red) and in the control group (blue) using principal component analysis (PCA), uniform manifold approximation and projection (UMAP) and t-distributed stochastic neighbor embedding (t-SNE). D) PCA variable graph showing the correlations of the clinical variables with the first two principal components E) PCA variable graph showing the correlations of all proteins with the first two principal components F) Correlations between clinical variables and the first seven principal components (PC1-7, accounting for > 40 % of the total proteomic variation) in the PCA. The order of clinical variables is based on the strength of the absolute correlation coefficient with PC1-7, the highest correlation shown to the left.

**Supplemental Figure S2.** Pathway overrepresentation analysis using the databases Jensen Diseases (green) and GO Biological Processes (red) in Enrichr (Kuleshov et al. Nucleic Acids Research. 2016; gkw3771). The x-axes show the negative logarithm of the adjusted p-value for each of the top ten pathways. Vertical line indicates adjusted p = 0.05.

**Supplemental Figure S3.** Correlation matrix of the top 30 diabetes-associated proteins versus clinical variables. Numbers within the matrix represent r-values from spearman correlations, colored if FDR-adjusted p-value <0.05.

**Supplemental Figure S4.** Relative concentrations (NPX units) at all timepoints for the five proteins with the most significant changes during 3 months of diabetes treatment.
